# Anti-Entry Activity of Natural Flavonoids against SARS-CoV-2 by Targeting Spike RBD

**DOI:** 10.3390/v15010160

**Published:** 2023-01-04

**Authors:** Jie-Ru Meng, Jiazheng Liu, Lu Fu, Tong Shu, Lingzhi Yang, Xueji Zhang, Zhi-Hong Jiang, Li-Ping Bai

**Affiliations:** 1 State Key Laboratory of Quality Research in Chinese Medicine, Macau Institute for Applied Research in Medicine and Health, Guangdong-Hong Kong-Macao Joint Laboratory of Respiratory Infectious Disease, Macau University of Science and Technology, Taipa 999078, China; 2 School of Biomedical Engineering, Health Science Center, Shenzhen University, Shenzhen 518060, China

**Keywords:** flavonoid, antiviral activity, SARS-CoV-2, structure–activity relationship, viral entry inhibitor

## Abstract

COVID-19 is still a global public health concern, and the SARS-CoV-2 mutations require more effective antiviral agents. In this study, the antiviral entry activity of thirty-one flavonoids was systematically evaluated by a SARS-CoV-2 pseudovirus model. Twenty-four flavonoids exhibited antiviral entry activity with IC_50_ values ranging from 10.27 to 172.63 µM and SI values ranging from 2.33 to 48.69. The structure–activity relationship of these flavonoids as SARS-CoV-2 entry inhibitors was comprehensively summarized. A subsequent biolayer interferometry assay indicated that flavonoids bind to viral spike RBD to block viral interaction with ACE2 receptor, and a molecular docking study also revealed that flavonols could bind to Pocket 3, the non-mutant regions of SARS-CoV-2 variants, suggesting that flavonols might be also active against virus variants. These natural flavonoids showed very low cytotoxic effects on human normal cell lines. Our findings suggested that natural flavonoids might be potential antiviral entry agents against SARS-CoV-2 *via* inactivating the viral spike. It is hoped that our study will provide some encouraging evidence for the use of natural flavonoids as disinfectants to prevent viral infections.

## 1. Introduction

In the last twenty years, coronaviruses have caused three epidemic diseases with a significant mortality rate [1]. Of these, COVID-19, caused by the novel coronavirus in 2019, is still affecting the world today as a result of the continuing emergence of more aggressive mutated strains [2,3]. As of now, there have been 645 million confirmed cases of COVID-19 worldwide, including 6.64 million deaths [4]. While progress has been made in the development of vaccines and antiviral drugs, the increasing number of infections, the uneven global distribution of vaccines and the emergence of new SARS-CoV-2 variant strains necessitate the ongoing search for potential antiviral agents [5,6,7]. Natural products are an attractive source of antiviral agents in the face of a challenging drug development process [8,9,10]. Based on PubMed, over 15,000 papers have been addressed on the natural products with COVID-19 research since the emergence of the SARS-CoV-2. Many natural products were found to have potential as anti-SARS-CoV-2 agents based on the in silico virtual screening, in vitro and in vivo studies [11,12,13].

**Figure 1:**
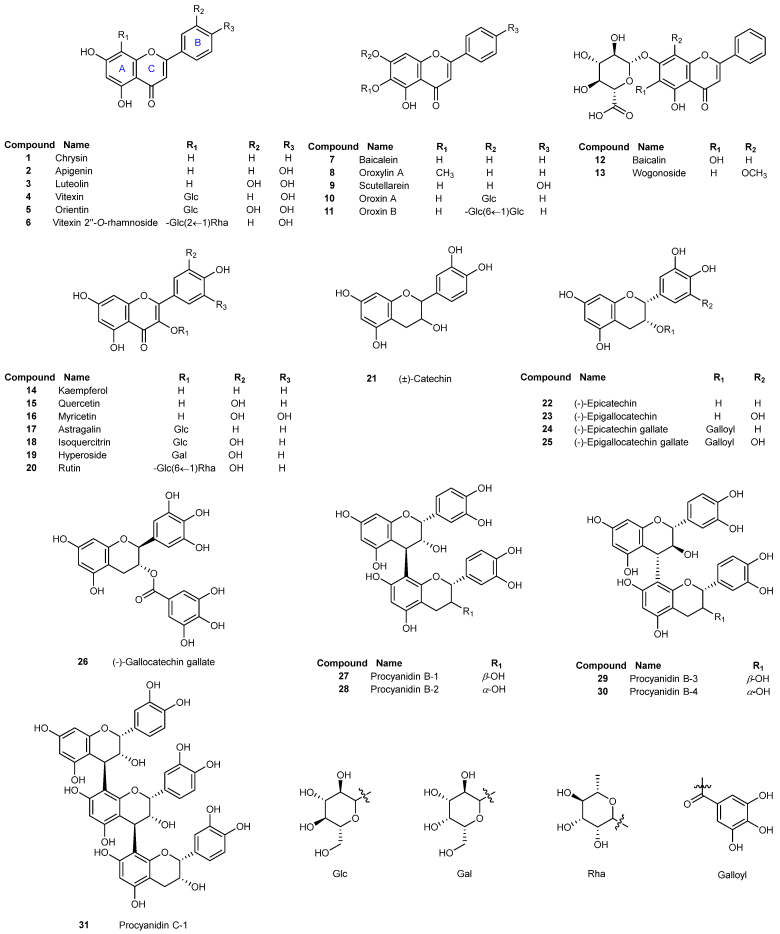
Chemical structures of the tested flavonoids.

Flavonoids are a large group of phytochemicals commonly derived from medicinal plants, fruits and vegetables. Its chemical structure is formed by two benzene rings (A and B rings) linked by a heterocyclic pyrene ring (C ring) and contains multiple phenolic hydroxyl groups. Based on their chemical structure, flavonoids can be divided into several subgroups including flavone, flavonol, flavanol, and so on. Flavonoids possess diverse pharmacological activities, including antimicrobial, anti-oxidant, anti-inflammatory, anticancer and antiviral activities [14,15,16]. Additionally, flavonoids are relatively safe associated with low toxicity and have the ability to act synergistically with other drugs [17]. Therefore, flavonoids are being increasingly studied in the development of anti-SARS-CoV-2 agents [18,19,20,21]. Numerous studies have shown that flavonoids exhibit anti-SARS-CoV-2 effect by inhibiting multiple targets and steps in the viral life cycle, including inhibition of viral entry, viral mRNA, viral proteases and viral replication in a direct antiviral manner, and also indirectly by affecting interferons and pro-inflammatory cytokines [22,23,24,25,26,27]. Among them, it is regarded as one of the most critical steps to prevent infection by SARS-CoV-2 that is the interaction between SARS-CoV-2 spike receptor binding domain (RBD) and the human ACE2 receptor during the viral entry [28]. Several studies have shown that some flavonoids have the ability to disrupt spike–ACE2 interaction [29,30,31]. For instance, the isorhamnetin was found to inhibit SARS-CoV-2 spike pseudovirus entering HEK-293-ACE2 cells in vitro [32]. The oroxylin A was discovered to block the entrance of SARS-CoV-2 into HEK-293T-ACE2 cells by specifically binding to ACE2 receptor [33]. The epigallocatechin-3-gallate (EGCG) was also reported to significantly inhibit SARS-CoV-2 and other coronavirus infections by blocking the spike–ACE2 interaction [34]. Similarly, our group have found that baicalein and scutellarein (bioactive flavonoids of *Scutellariae Radix*) inhibited viral entry by targeting the interaction of viral spike with ACE2 receptor [35]. All the above findings suggested that different subclasses of flavonoids have the potential as SARS-CoV-2 entry inhibitors. Nevertheless, the reported antiviral activities of some flavonoids in the previous studies were scattered and incomparable, due to the different bioassay methods and varied experimental conditions. Up to now, no systematic investigation and comparison have been conducted for various subclasses of flavonoids on the inhibition of SARS-CoV-2 viral entry into host cells. Therefore, the present study aims to evaluate the antiviral entry activity of a panel of flavonoids (Figure 1) including flavones, flavonols and flavanols, and summarize their structure–activity relationship as viral entry inhibitors. For this purpose, a well-established SARS-CoV-2 pseudovirus assay was utilized to test antiviral entry effect of flavonoids. Biolayer interferometry (BLI) binding assay and molecular docking study were also used to measure the binding affinities and predict the binding modes of selected bioactive flavonoids with SARS-CoV-2 spike RBD protein. It is hoped that the obtained structure–activity relationship may provide guidance to design and synthesize new effective flavonoid-based antiviral entry molecules against SARS-CoV-2 in the near future.

## 2. Materials and Methods

### 2.1. Materials and Reagents

All flavonoid standards were provided by Chengdu Must Bio-Technology Co., Ltd (Chengdu, China). Cepharanthine was bought from Macklin (Shanghai, China). The purity of all compounds was more than 98%. The stock solution (50 mM) was prepared with dimethyl sulfoxide (DMSO). The SARS-CoV-2 (2019-nCoV) spike RBD-His recombinant protein was obtained from Sino Biological (Beijing, China). The plasmid of pNL4-3.Luc.R-E- was bought from the NIH AIDS repository. The plasmid of pcDNA3.1-SARS-CoV-2-Spike-Myc, Firefly luciferase reporter gene assay kit, hygromycin B and Lipofectamine™ 8000 (Lipo8000) transfection reagent were bought from Beyotime Biotechnology (Shanghai, China). The nickel-charged nitrilotriacetic acid (Ni-NTA) biosensors were acquired from Fortebio (San Jose, CA, USA).

### 2.2. Cell Culture

HEK-293T cells, LO2 cells, Beas-2B cells and HEK 293 cells were purchased from American Type Culture Collection (ATCC, Bethesda, MD, USA). HEK-293T cells highly expressing ACE2 (HEK-293T-ACE2^h^ cells) were constructed by Sino Biological (Beijing, China). Dulbecco modified eagle medium (DMEM), Opti-MEM, fetal bovine serum (FBS) and penicillin-streptomycin-glutamine mixture were obtained from Gibco (Grand Island, NY, USA). All the cell lines were cultured in DMEM supplemented with 10% FBS and 1% penicillin-streptomycin-glutamine mixture at 37 °C in humidified air containing 5% CO_2_. In addition, hygromycin B was used for the HEK-293T-ACE2^h^ cell culture media at a concentration of 100 µg/mL.

### 2.3. Cytotoxicity Assay

The cell viability was performed by MTT assay. Briefly, the HEK-293T-ACE2^h^ cells were plated in 96-well culture plates at a density of 1 × 10^4^ cells/well, while LO2, Beas-2B and HEK 293 cells were seeded in 96-well plates at a density of 5 × 10^3^ cells/well. The plates were incubated at 37 °C under 5% CO_2_ for 24 h to obtain a monolayer culture. Then, HEK-293T-ACE2^h^ cells were exposed to the tested compound at different concentrations or 0.1% DMSO as a control for 4 h to obtain the values of both CC_0_ and TC_50_, while LO2, Beas-2B and HEK 293 cells were treated for 24 h to calculate the IC_50_ values. After treatment, 10 μL of MTT reagent (5 mg/mL) was added into each well and the cells were incubated at 37 °C in the CO_2_ incubator for another 4 h. Subsequently, the supernatant was removed. The purple crystal formazan was then dissolved in 100 μL of DMSO by oscillation. The absorbance values were measured by SpectraMax Paradigm multi-mode detection platform (Molecular Devices, San Jose, CA, USA) at a wavelength of 570 nm. All the experiments were repeated in triplicate.

### 2.4. Evaluation of SARS-CoV-2 Spike Pseudotyped Virus Entry into ACE2^h^ Cells

SARS-CoV-2 pseudovirus was prepared by the same approach as previously published [35,36,37,38]. The spike protein expression plasmid (pcDNA3.1-SARS-CoV-2-Spike-Myc) was co-transfected with the HIV backbone vector pNL4-3.Luc.R-E- into HEK-293T cells by the Lipo8000 transfection reagent. The SARS-CoV-2 pseudoviruses bearing the Firefly luciferase were harvested 24 h after transfection. The inhibitory activity of flavonoids against pseudovirus entry into host cells was then measured by quantification of the luciferase activity. In brief, the HEK-293T-ACE2^h^ cells were seeded in 96-well plates at a density of 1 × 10^4^ cells. After 24 h of incubation, the 100 μL medium containing a certain dose of the compound was first added into the cells and the cells were incubated for 2 h. The host cells were then infected for 2 h with the 100 μL mixture solution of pseudovirus and compound (consisted of 30 μL pseudovirus, 20 μL media, and 50 μL two-fold concentration of compounds). After 2 h of pseudovirus infection, the culture medium containing the virus was removed and replaced by 100 μL of fresh DMEM. Subsequently, the cells were incubated continuously at 37 °C for 48 h. The culture supernatant was removed gently, and 100 μL of cell lysate was added to each well to lyse cells. After being shaken for 10 min, 100 μL of cell lysate was transferred to the well of white solid 96-well plates, and 100 μL luminescence solution was then added to each well. The luciferase luminescence was measured at 568 nm by a microplate reader (SpectraMax iD5 multi-mode microplate reader, Molecular Devices, San Jose, CA, USA) immediately. Cells without viruses and compounds were used as the blank control, and cells with only viruses were utilized as the pseudovirus control. The values of luciferase luminescence of the pseudovirus control group were defined as 100%, and the luminescence values of compound-treated groups were normalized accordingly. The percentage of pseudovirus inhibition was calculated as 100% − (sample signals − blank control signals)/(virus control signals − blank control signals) × 100%. The IC_50_ value was determined using the GraphPad Prism 8.0 software (La Jolla, CA, USA). All measurements were conducted in triplicate from three independent experiments. The data were presented as the means ± standard deviations (SDs).

### 2.5. Biolayer Interferometry (BLI) Binding Assay

The binding kinetics of flavonoids with SARS-CoV-2 spike RBD protein were analyzed by biolayer interferometry on an Octet system (Octet RED96, ForteBio, Fremont, CA, USA). Initially, a histidine-tagged SARS-CoV-2 spike RBD protein (25 μg/mL aqueous solution) was immobilized on Ni-NTA biosensors by a protein-loading program of the instrument. Each stock solution of samples (50 mM in DMSO) was serially diluted by PBS buffer with a final DMSO concentration of 0.4%. Both protein-immobilized and empty biosensors were equilibrated in PBS buffer for 10 min at room temperature before preceding data acquisition, and all experiments were performed at 30 °C. Both the protein-coated and empty biosensors were dipped in wells containing serially diluted samples. The signal of background was subtracted from all samples by dipping a protein-immobilized biosensor in the blank buffer. Under the same conditions, empty biosensors were also dipped in serially diluted samples for reference subtraction. The subtracted sensorgrams were then fitted to a 1:1 binding model by using Octet Data Analysis Software v11.1 (ForteBio) to calculate the equilibrium dissociation constant (K_D_) values for the interaction.

### 2.6. Molecular Docking Study

The molecular docking studies were performed using the Discovery Studio, a peer-reviewed molecular simulation workstation that integrated a variety of computer-aided drug design technologies. The spike RBD protein of wild type SARS-CoV-2 (PDB ID: 6M0Z) was downloaded from the RCSB PDB database. The protein was removed the water molecules and completed the missing amino acids using Discovery Studio 2021, and then saved in “.pdb” format. The druggable binding sites of spike RBD protein were predicted using the ProteinsPlus web server (Available online: https://proteins.plus, accessed on 7 September 2022) [39,40]. The 3-dimensional (3D) structures of kaempferol, quercetin and myricetin were downloaded from PubChem in “.sdf” format, and the energies of three compounds were minimized using the CHARMM force field. Then, semi-flexible CDOCKER program was employed to dock and analyze the interaction between three compounds (ligand) and spike RBD protein (receptor). In addition, the spike RBD structures of different SARS-CoV-2 variants (Delta, Omicron BA.1 and BA.2) were also downloaded and subjected to the same pretreatment according to the above approach. The spike RBD proteins from different strains were processed for sequence alignment and protein superimposition by the “Align Structures” module of Discovery Studio (version 2021, BIOVIA Corp, San Diego, CA, USA). Afterwards, the semi-flexible CDOCKER program was still utilized to dock kaempferol, quercetin and myricetin with the druggable binding sites of spike RBD proteins from different mutant strains. Finally, the results collected from the docking simulation were visualized and analyzed by Discovery Studio 2021 software.

### 2.7. Statistical Analysis

The statistical analysis was conducted by GraphPad Prism 8.0.2 software (La Jolla, CA, USA). The comparison between two sets of data was performed via independent samples *t*-test. The *p*-values of 0.05 or less were considered statistically significant. Typically, data from at least three independent experiments were used for analyses, and all data were expressed as the mean ± SD.

## 3. Results and Discussion

### 3.1. Inhibitory Activities of Flavonoids against the Entry of SARS-CoV-2 Pseudovirus into Host Cells and Structure–Activity Relationship Analysis

Thirty-one flavonoids (Figure 1) were firstly evaluated for their inhibitory activity against SARS-CoV-2 entry into host cells using a well-established SARS-CoV-2 pseudovirus assay [35,36,37,38]. Cepharanthine, a reported inhibitor of viral entry, was used as a positive control [41,42]. Initially, the maximum nontoxic concentration (CC_0_) values and median toxic concentration (TC_50_) values of all tested flavonoids were determined by MTT assay against the host cells of HEK-293T-ACE2^h^. As displayed in Table 1, all the tested flavonoids exhibited low cytotoxicity on the host cells compared to the positive control, cepharanthine [41,42]. The CC_0_ values of tested flavonoids were greater than 100 μM except for apigenin (**2**), indicating that flavonoids had no cytotoxic effect on HEK-293T-ACE2^h^ cells under the tested concentration of 100 μM. Subsequently, the antiviral activity of flavonoids was evaluated under doses of no higher than the corresponding CC_0_ values. The IC_50_ values of flavonoids preventing SARS-CoV-2 pseudovirus from entry into host cells and the values of selectivity index (SI) defined as the ratio of TC_50_/IC_50_ were shown in Table 1. Most of the tested flavonoids showed inhibitory activity against pseudovirus entry into the host cells with IC_50_ values ranging from 10.27 to 172.63 μM and SI values of 2.33 ~ 48.69. Eight flavonoids, luteolin (**3**), oroxylin A (**8**), scutellarein (**9**), kaempferol (**14**), quercetin (**15**), myricetin (**16**), (-)-epigallocatechin gallate (**25**) and (-)-gallocatechin gallate (**26**) potently suppressed the entry of SARS-CoV-2 pseudovirus into host cells with IC_50_ values less than 50 μM. Myricetin exhibited the most potent antiviral activity with an IC_50_ value of 10.27 ± 2.32 µM and an SI value more than 48.69 among all the tested flavonoids. Moreover, myricetin did not demonstrate any cytotoxicity to the host cells under the tested concentration greater than 500 μM. Cepharanthine, the reported viral entry inhibitor showed more potent antiviral entry activity with an IC_50_ value of 1.30 ± 0.18 µM but displayed much stronger cytotoxicity on the host cells with a TC_50_ value of 11.54 μM. Most of the tested flavonoids exhibited almost no cytotoxicity at a high dosage of 500 μM, and four flavonoids (luteolin, quercetin, myricetin and EGCG) showed much higher SI values (>15.99) than that (8.88) of cepharanthine. The SI value is a ratio between the toxicity and the antiviral effect of a compound and commonly used for comparing the antiviral efficacy of tested compounds [43]. The higher the SI value is, the more efficient and safer the drug is in preventing viral infection. Taken together, these findings indicated that some flavonoids are promising and safe antiviral entry candidates.

**Figure 2:**
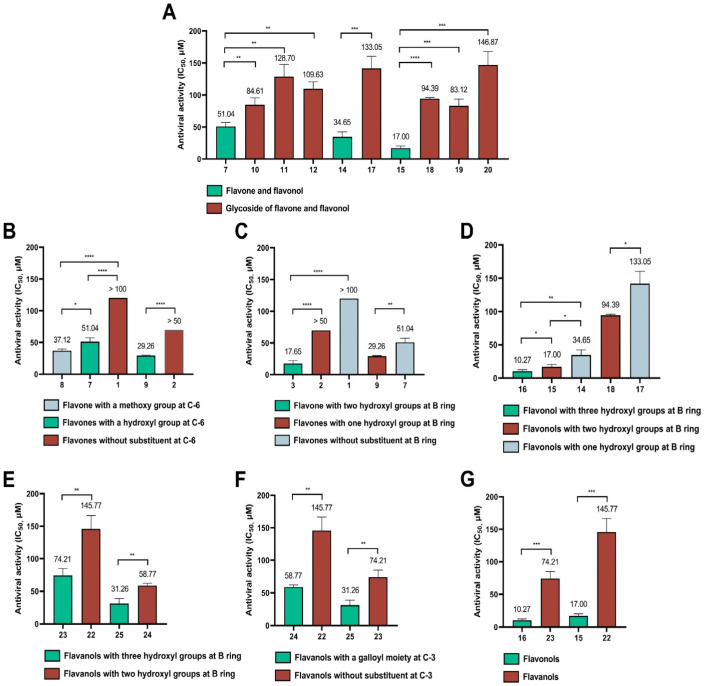
Comparison of inhibitory activity of flavonoids against SARS-CoV-2 pseudovirus entry into host cells. (**A**) Effect of glycosyl moieties on flavonoids; (**B**) Effect of a hydroxyl or methoxy group at C-6 on flavones; (**C**) Impact of the number of hydroxyl groups at B ring on flavones; (**D**) Impact of the number of hydroxyl groups at B ring on flavonols; (**E**) Impact of the number of hydroxyl groups at B ring on flavanols; (**F**) Effect of a galloyl moiety at C-3 on flavanols; (**G**) Effect of a planar C ring of flavonols compared to a stereo C ring of flavanols. Data were obtained from three separate experiments. * *p* < 0.05, ** *p* < 0.01, *** *p* < 0.001, **** *p* < 0.0001.

In order to further investigate the key pharmacophores of flavonoids contributing to the antiviral entry activity, we also analyzed the structure–activity relationship (SAR) of a series of grouped or paired flavonoids (Figure 2).

Firstly, glycosylation decreased the antiviral entry activities of flavonoids. As shown in Figure 2A, the presence of a glycosyl moiety at the C-7 position (**10–12**) reduced the inhibitory activity against pseudovirus. The activity of both oroxin A (**10**) with glucosyl substitution and baicalin (**12**) with glucuronic acid substitution were significantly reduced compared to their aglycone (baicalein). Particularly, the presence of the two glycosyl groups in oroxin B (**11**) resulted in a more than 50% decrease in antiviral activity of oroxin B (**11**) compared to baicalein (**7**). Furthermore, a similar SAR was also observed among flavonols with a glycosyl substitution at C-3. The inhibitory activity of flavonol was also stronger than that of flavonol glycoside. By comparison of astragalin (**17**) and its corresponding aglycone of kaempferol (**14**), it was found that glycosylation of the hydroxyl group at the C-3 position diminished inhibitory potency significantly (*p* < 0.001). Meanwhile, quercetin (**15**) showed 4.9–8.6 folds stronger inhibitory activity than its glycosides of isoquercitrin (**18**), hyperoside (**19**) and rutin (**20**). Consequently, glycosyl groups at both C-3 and C-7 had considerably negative influence on the antiviral entry activity of flavonoids.

Secondly, a substituent at the C-6 position enhanced the antiviral entry activity. As depicted in Figure 2B, it could be concluded from the IC_50_ values that both hydroxy substitution and methoxy substitution at C-6 increased the antiviral activity as evidenced from the following. The antiviral entry activity of oroxylin A (**8**) was at least 2.7-fold more potent than that of chrysin (**1**). The hydroxyl group in both baicalein (**7**) and scutellarein (**9**) led to a significant increase of the antiviral activities compared to chrysin (**1**) and apigenin (**2**), respectively. The SI values of baicalein (**7**), oroxylin A (**8**) and scutellarein (**9**) were also higher than those of chrysin (**1**) and apigenin (**2**), suggesting that flavonoids (**7–9**) had safer and better drug efficacy. In addition, comparison of the antiviral activities of oroxylin A (**8**) and baicalein (**7**), indicated that a methoxy substituent displayed a better antiviral activity than a hydroxyl substituent at C-6.

Thirdly, the number of hydroxyl groups at B ring of the flavonoids significantly contributed to the antiviral entry activity against SARS-CoV-2 pseudovirus. As summarized in Figure 2C, luteolin (**3**) with *o*-diphenol hydroxyl groups at B ring showed much higher antiviral entry activity than apigenin (**2**) bearing just one hydroxyl group at B ring and chrysin (**1**) without hydroxyl group at B ring. Likewise, baicalein (**7**) which differed structurally from scutellarein (**9**) only in the absence of the hydroxyl group at C-4′, showed weaker antiviral activity than scutellarein (**9**). As shown in Figure 2D, myricetin bearing 3′, 4′, 5′-trihydroxy at B ring (**16**) showed the most potent antiviral activity against SARS-CoV-2 pseudovirus with an IC_50_ value of 10.27 μM, followed by quercetin containing 3′, 4′-dihydroxyl at B ring (**15**) with an IC_50_ value of 17.00 μM, and kaempferol with only 4′-hydroxyl at B ring (**14**) exhibited a relatively weaker activity with an IC_50_ value of 34.65 μM. This indicated that the increased number of hydroxyl groups at its B ring enhanced antiviral entry activity of flavonols. The same SAR was also observed between astragalin (**17**) and isoquercitrin (**18**). A hydroxyl group at the C-3′ position increased the antiviral activity of isoquercitrin (**18**) compared to that of any substitution of astragalin (**17**). The importance of hydroxyl groups at B ring for antiviral activity was further supported by the evidence from flavanols (Figure 2E). The presence of a hydroxyl group at C-5′ of EGC (**23**) and EGCG (**25**) was responsible for their higher antiviral activity than EC (**22**) and ECG (**24**), respectively. Overall, the substitution of hydroxyl groups at B ring contributed to the antiviral activity of flavonoids. The activity seemed to depend largely on the number of hydroxy groups at B ring, which might be related to the interaction with target proteins.

Fourthly, a galloyl moiety at C-3 of flavanols was beneficial to enhance the antiviral activity against SARS-CoV-2 pseudovirus. As shown in Figure 2F, the antiviral activity of ECG (**24**) was 2.5-fold stronger than that of EC (**22**), and EGCG (**25**) also showed 2.3-fold more potent antiviral entry activity than EGC (**23**).

Finally, the antiviral activities of flavonols were stronger than those of the corresponding flavanols (Figure 2G). Based on the comparison of antiviral activity between the flavanols and flavonols, it was found that a planar C ring formed by a double bond at C2-C3 and a carbonyl group at C-4 is crucial for the antiviral activity of flavonols compared to a stereo C ring of flavanols. For instance, myricetin (**16**) was 7.2-fold more potent antiviral entry into host cells than EGC (**23**) (*p* < 0.001), and quercetin (**15**) displayed 8.6-fold stronger antiviral activity than EC (**22**) (*p* < 0.001). Furthermore, both quercetin (**15**) and myricetin (**16**) had higher SI values than EC (**22**) and EGC (**23**), indicating that these two flavonols may have broad safety margins and good application prospects. These results confirmed that the flavonol skeleton is more advantageous to the antiviral activity against SARS-CoV-2 pseudovirus entry into host cells.

**Figure 3:**
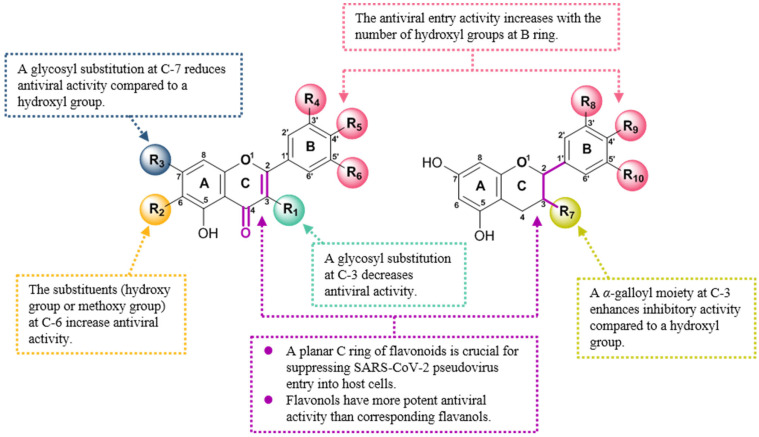
Summary of the structure–activity relationship of flavonoids as SARS-CoV-2 entry inhibitors.

Taken together, the summary of SAR for flavonoids as viral entry inhibitors was shown in Figure 3. The antiviral activity increased with the number of hydroxyl groups at B ring for both flavonols and flavanols. The planar C ring was critical for the antiviral entry activity of flavonoids. Additionally, both hydroxyl and methoxy groups at C-6 were favored to antiviral activity of flavones. The presence of a galloyl group at C-3 increased the antiviral activity of flavanols. However, glycosylation at both C-3 and C-7 significantly decreased the antiviral activity of flavones and flavonols.

### 3.2. Binding Affinities of Selected Antiviral Entry Flavonoids with SARS-CoV-2 Spike RBD Protein

**Figure 4:**
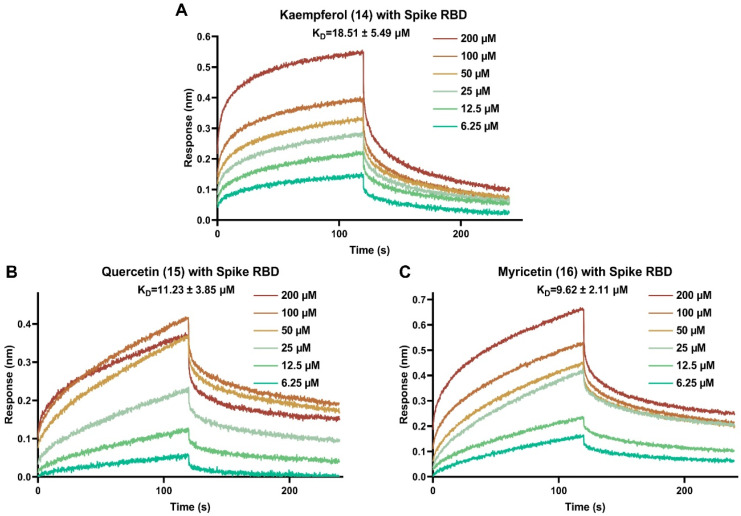
Binding curves of selected bioactive flavonoids with SARS-CoV-2 spike RBD by BLI binding kinetics assay. (**A**) The interaction of kaempferol with viral spike RBD protein; (**B**) the interaction of quercetin with viral spike RBD protein; (**C**) the interaction of myricetin with viral spike RBD protein.

Binding of the viral spike to the ACE2 receptor of the host cells initiates viral invasion. The interaction between SARS-CoV-2 spike RBD and host ACE2 receptor is the first key step to block the viral entry into the host cells. Through SARS-CoV-2 pseudovirus assay, it was found that three flavonols with varying hydroxyl groups at B ring (kaempferol, quercetin and myricetin) have good potential to inhibit the entry of SARS-CoV-2 pseudovirus into host cells, that is, these three flavonols might affect the interaction between SARS-CoV-2 spike RBD and human ACE2 receptor. Therefore, these effective antiviral entry inhibitors were selected as representative flavonoids for further binding interaction analysis with viral spike RBD protein. BLI is a real-time detection method for bio-molecular interaction, which can detect whether compounds directly interact with viral spike RBD. As displayed in Figure 4, kaempferol (**14**), quercetin (**15**) and myricetin (**16**) dose-dependently bound to SARS-CoV-2 spike RBD. All of the three flavonols bound strongly to SARS-CoV-2 spike RBD with K_D_ values of 18.51 ± 5.49, 11.23 ± 3.85 and 9.62 ± 2.11 μM, respectively. The binding signals were strong, especially myricetin (**16**), which reached up to 0.60 nm at the concentration of 200 μM. The findings revealed that these effective antiviral entry flavonoids could effectively prevent SARS-CoV-2 from binding to the host’s ACE2 receptor by blocking viral spike RBD, thereby inhibiting viral entry into the host cells.

### 3.3. Identification of Potential Binding Pockets and Binding Mode for Bioactive Flavonoids Targeting SARS-CoV-2 Spike RBD

**Figure 5:**
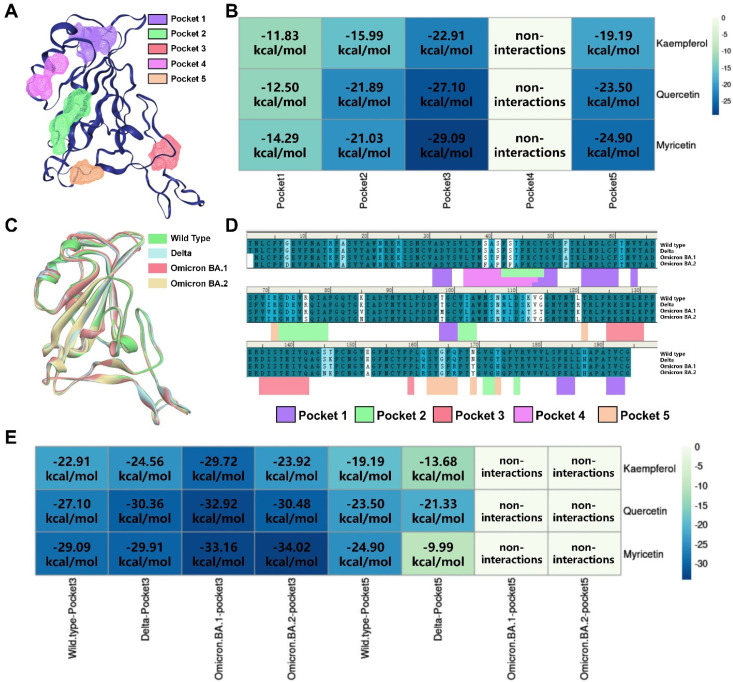
Prediction of the binding models of flavonols by computational modeling study. (**A**) Possible binding sites of SARS-CoV-2 spike RBD identified by DoGSiteScorer. (**B**) Heatmap of molecular docking scores for flavonols. (**C**) The 3D structure superimposition of spike RBD from different mutant strains. (**D**) Align sequence of spike RBD from different mutant strains. (**E**) Heatmap of molecular docking scores of Pocket 3 and Pocket 5 from different mutant strains for flavonols, supporting that Pocket 3 might be the key druggable binding site for flavonols to inhibit SARS-CoV-2 infection.

To further investigate the binding mode of active flavonols to the viral spike target, the DoGSiteScorer method of ProteinsPlus website was used to calculate the druggable binding sites of SARS-CoV-2 spike RBD. The selection and definition of docking pockets are critical for molecular simulation studies. DoGSiteScorer is a grid-based method that utilizes differential Gaussian filter techniques to detect potential pockets and binding sites based on the 3D structure of the protein. As shown in Table 2 and Figure 5A, there are five potential binding sites on the SARS-CoV-2 spike RBD. Then, we docked kaempferol, quercetin and myricetin with each of these five active binding sites using a semi-flexible docking program, CDOCKER based on kinetic simulation. Based on CDOCKER energy scores (Figure 5B), we found that Pocket 3 and Pocket 5 are the key active sites for flavonols binding to spike RBD. It is worth mentioning that Gln498, Asn501 and Tyr505 amino acid residues located in Pocket 5 have been found to be the key amino acid residues for SARS-CoV-2 infection to human host cells. Meanwhile, it is suggested that Pocket 3 might be a potential allosteric binding site in the spike RBD.

**Figure 6:**
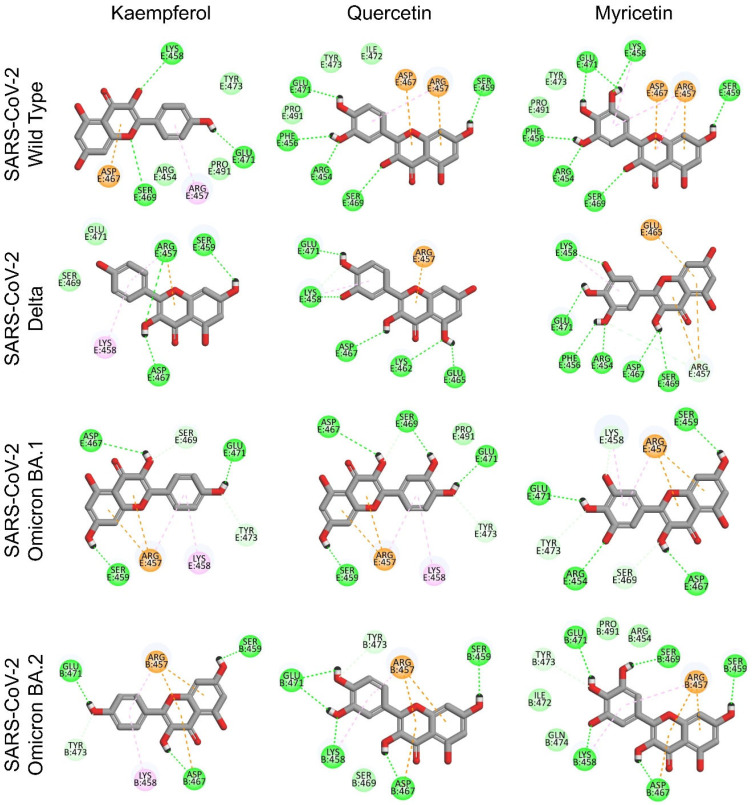
Molecular models of flavonols binding to the Pocket 3 in spike RBD from different mutant strains.

The ongoing mutation of SARS-CoV-2 strains is an important reason for the global pandemic. By comparing the SARS-CoV-2 spike RBD protein sequences of wild type, Delta, Omicron BA.1 and BA.2 (Figure 5C,D), it was found that the amino acids constituting the Pocket 5 region are highly susceptible to mutations, while the amino acid residues constituting the Pocket 3 region are highly conserved. The kaempferol, quercetin and myricetin were then docked into Pocket 3 and Pocket 5 of different strains, respectively. As shown in Figure 5E, with the mutation of the virus strains, G496S, Q498R, N501Y and Y505H mutations of Pocket 5 gradually increased the binding free energies of flavonols with Pocket 5. This finding suggested that the binding ability of flavonols to Pocket 5 might be decreased with strain mutation and explained the reason why drugs designed for spike RBD were prone to off-target. However, Pocket 3 is highly conserved in virus mutations, with no amino acid changes in the binding region. The binding free energies of flavonols to Pocket 3 showed a tendency to decrease with virus mutation, suggesting that the binding ability of flavonols to Pocket 3 might have the potential to overcome the mutation of SARS-CoV-2. In addition, the binding free energies of flavonols with Pocket 3 are lower than that of Pocket 5, suggesting that Pocket 3 might be the key druggable binding sites for flavonols to inhibit SARS-CoV-2 infection. We then analyzed the flavonol binding pattern to the Pocket 3 (Figure 6) and found that both the A and C rings of flavonols were the key pharmacophores for binding with the Pocket 3 in different SARS-CoV-2 variants. The hydroxyl group in the C-3 position on the flavonol C ring could form a stable hydrogen bond with Pocket 3 and remain stably inside Pocket 3. This discovery also explained why flavonol aglycones (**14** and **15**) had a stronger antiviral entry ability against SARS-CoV-2 than corresponding glycosides (**17** and **18–20**, respectively), which was consistent with the above structure–activity relationship (Figure 2A) based on the pseudovirus assay.

### 3.4. Cytotoxic Evaluation of Flavonoids in Human Normal Cell Lines

The ideal antiviral entry agents should prevent virus entry without cytotoxicity toward the host normal cells. Therefore, bioactive flavonoids with antiviral activity were further tested for their cytotoxic activity against human normal cell lines using the MTT assay. As shown in Table 3, these flavonoids exhibited no significant cytotoxicity against the tested human normal cell lines with IC_50_ values greater than 200 μM, except for luteolin (**3**) which showed an IC_50_ value of 182.68 μM to LO2 cells. In particular, the human lung epithelial cell line Beas-2B, which expressed ACE2, is susceptible to SARS-CoV-2 infection [44]. The positive control, cepharanthine, showed considerable cytotoxicity against Beas-2B cells with an IC_50_ value of 26.13 μM, while myricetin (**16**) as the most effective antiviral entry flavonoid in this study did not show any significant cytotoxic effect on all three cell lines with tested dose of 500 μM. The cytotoxicity of the tested flavonoids was much lower than cepharanthine toward all three human normal cell lines. These findings suggested that these flavonoids with low cytotoxicity might be harmless for humans and are worth further study as antiviral inhibitors.

The cytotoxic effects were measured using MTT assay. Cells were treated with flavonoids for 24 h. IC_50_ values were determined using GraphPad Prism 8.0 software. All the data were expressed as means ± standard deviations (SDs) from three independent experiments.

## 4. Discussion

Natural flavonoids are phytochemicals found in medicinal plants, soybeans, teas, fruits, and vegetables. In this investigation, the subclasses of representative flavone, flavonol, and flavanol were tested for antiviral entry activity against SARS-CoV-2 using a well-established pseudovirus system, BLI assay, and molecular docking study. As SARS-CoV-2 entry inhibitors, twenty-four flavonoids have antiviral potential, with myricetin being the most effective (IC_50_ value of 10.27 M against SARS-CoV-2 pseudovirus). Molecular docking also revealed that three flavonols (myricetin, quercetin, and kaempferol) have the potential to inhibit SARS-CoV-2 variants by targeting the conserved Pocket 3 of the spike RBD. The antiviral entry effect of these three flavonols on various SARS-CoV-2 variants needs to be further validated in a biosafety level 3 laboratory.

Some isoflavones, such as genistein from soybean, are dietary phytoestrogens that have recently been shown to protect mice from hydrochloric acid-induced pulmonary fibrosis [45]. Lung fibrosis is a long-term side effect of SARS-CoV-2 infection. Natural flavonoids, which are safe for humans, according to our findings and the published literature [45], may play important roles in the prevention and treatment of COVID-19 [35].

## 5. Conclusions

In this study, the antiviral entry activity of natural flavonoids was systematically evaluated using a SARS-CoV-2 spike pseudovirus system. Most of the tested flavonoids showed antiviral entry activity. The structure–activity relationship of flavonoids as viral entry inhibitors was extensively investigated and revealed that a planar C ring formed by a double bond at C2–C3 and a carbonyl group at C-4 is critical for the antiviral entry activity of flavonols. The number of hydroxyl groups at B ring plays an important role in increasing antiviral activity of flavonoids. This is the first report of a comprehensive and systematic study on SAR of flavonoids as viral entry inhibitors. Additionally, BLI and molecular docking studies suggested that these flavonoids might prevent viral infection by blocking viral spike RBD’s interaction with ACE2 receptor. The molecular docking also revealed that flavonols could stably bind to the Pocket 3, the non-mutant regions of SARS-CoV-2 variants, implying that flavonols may also have inhibitory activity against mutant strains. As development of anti-SARS-CoV-2 drugs is a lengthy process, it is more important to prevent viral infection than to treat the disease. Therefore, natural flavonoids with low toxicity could be applied in a variety of ways to prevent viral infections, such as air sprays, disinfectants or hand sanitizers. It is hoped that our findings promote future studies on natural flavonoids as anti-infectious agents against SARS-CoV-2.

## Figures and Tables

**Table 1:** The cytotoxic effects of flavonoids on HEK-293T-ACE2^h^ cells and inhibitory activities against SARS-CoV-2 pseudovirus’ entrance into host cells.

No.	Flavonoids	CC_0_^a^ (μM)	TC_50_^b^ (μM)	IC_50_^c^ (μM)	SI ^d^
**1**	Chrysin	100	168.50 ± 12.06	>100	<1.69
**2**	Apigenin	50	90.88 ± 3.13	>50	<1.82
**3**	Luteolin	200	>300	17.45 ± 4.46	>17.19
**4**	Vitexin	300	>500	>300	ND
**5**	Orientin	300	>500	63.73 ± 9.61	>7.85
**6**	Vitexin-2″-*O*-rhamnoside	300	>500	>300	ND
**7**	Baicalein	200	>500	51.04 ± 6.48	>9.80
**8**	Oroxylin A	100	>300	37.12 ± 2.58	>8.08
**9**	Scutellarein	200	>300	29.26 ± 0.74	>10.25
**10**	Oroxin A	300	>300	84.61 ± 10.81	>3.55
**11**	Oroxin B	300	>300	128.70 ± 19.03	>2.33
**12**	Baicalin	300	>500	109.63 ± 11.02	>4.56
**13**	Wogonoside	300	>500	119.07 ± 21.00	>4.20
**14**	Kaempferol	200	>200	34.65 ± 7.69	>5.77
**15**	Quercetin	300	>500	17.00 ± 3.42	>29.41
**16**	Myricetin	300	>500	10.27 ± 2.32	>48.69
**17**	Astragalin	300	>500	133.05 ± 16.19	>3.76
**18**	Isoquercitrin	300	>500	94.39 ± 1.59	>5.30
**19**	Hyperoside	300	>500	83.12 ± 10.65	>6.02
**20**	Rutin	300	>500	146.87 ± 21.11	>3.40
**21**	(±)-Catechin	300	>500	156.80 ± 12.00	>3.19
**22**	(-)-Epicatechin (EC)	300	>500	145.77 ± 20.82	>3.43
**23**	(-)-Epigallocatechin (EGC)	200	453.10 ± 4.55	74.21 ± 10.72	6.11
**24**	(-)-Epicatechin gallate (ECG)	300	>500	58.77 ± 3.25	>8.51
**25**	(-)-Epigallocatechin gallate (EGCG)	300	>500	31.26 ± 7.40	>15.99
**26**	(-)-Gallocatechin gallate (GCG)	300	>500	43.41 ± 2.93	>11.52
**27**	Procyanidin B-1	300	>500	>300	ND
**28**	Procyanidin B-2	300	>500	>300	ND
**29**	Procyanidin B-3	300	>500	>300	ND
**30**	Procyanidin B-4	300	>500	151.37 ± 28.08	>3.30
**31**	Procyanidin C-1	300	>500	172.63 ± 20.74	>2.90
Positive control	Cepharanthine	6.25	11.54 ± 1.39	1.30 ± 0.18	8.88

^a^ CC_0_ means the maximum nontoxic concentration of flavonoids against the host cells of HEK-293T-ACE2^h^. ^b^ TC_50_ represents the concentration of flavonoids required to reduce HEK-293T-ACE2^h^ cell viability by 50%. ^c^ IC_50_ stands for the concentration of flavonoids that inhibited 50% pseudovirus entry into host cells. ^d^ SI was calculated as the ratio of TC_50_ to IC_50_. ND denotes not determined. All the data were expressed as means ± standard deviations (SDs) from three independent experiments.

**Table 2:** The parameters of possible druggable binding sites identified by DoGSiteScorer.

Number	Volume Å^3^	Drug Score	Site Center (X, Y, Z)
Pocket 1	216.45	0.59	−22.987082, 21.412573, 35.788383
Pocket 2	183.42	0.46	−24.487082, 22.662573, 19.788383
Pocket 3	181.95	0.45	−38.987082, 42.412573, 12.288383
Pocket 4	148.80	0.31	−24.987082, 17.662573, 31.288383
Pocket 5	109.63	0.16	−35.637121, 22.231293, 7.207497

**Table 3:** Cytotoxicity of flavonoids on human normal cell lines.

No.	Flavonoids	IC_50_ Values of Human Normal Cell Lines (μM)		
		Beas-2B	LO2	HEK 293
**3**	Luteolin	>300	182.68 ± 10.26	>300
**5**	Orientin	>500	>500	>500
**7**	Baicalein	493.75 ± 9.64	>500	>500
**8**	Oroxylin A	>300	>300	>300
**9**	Scutellarein	>300	>300	>300
**10**	Oroxin A	>300	>300	257.80 ± 10.57
**11**	Oroxin B	>300	>300	>300
**12**	Baicalin	>500	>500	>500
**13**	Wogonoside	>500	>500	>500
**14**	Kaempferol	>200	>200	>200
**15**	Quercetin	>500	>500	>500
**16**	Myricetin	>500	>500	>500
**17**	Astragalin	>500	>500	>500
**18**	Isoquercitrin	>500	>500	>500
**19**	Hyperoside	>500	>500	>500
**20**	Rutin	>500	>500	>500
**21**	(±)-Catechin	>500	>500	>500
**22**	(-)-Epicatechin (EC)	>500	>500	>500
**23**	(-)-Epigallocatechin (EGC)	369.00 ± 1.70	483.33 ± 1.52	>500
**24**	(-)-Epicatechin gallate (ECG)	>500	>500	>500
**25**	(-)-Epigallocatechin gallate (EGCG)	>500	430.40 ± 6.98	>500
**26**	(-)-Gallocatechin gallate (GCG)	>500	432.93 ± 8.96	>500
**30**	Procyanidin B-4	>500	>500	>500
**31**	Procyanidin C-1	>500	>500	>500
Positive control	Cepharanthine	26.13 ± 1.08	16.06 ± 0.13	24.30 ± 0.22

## Data Availability

The data supporting the findings of this investigation are available within the manuscript.
